# Reporting of endoscopy and histology of ulcerative colitis in routine clinical practice: How far we are!

**DOI:** 10.1002/ueg2.12432

**Published:** 2023-07-02

**Authors:** Fernando Magro, Maria Manuela Estevinho

**Affiliations:** ^1^ Unit of Pharmacology and Therapeutics Department of Biomedicine Faculty of Medicine University of Porto Porto Portugal; ^2^ Department of Biomedicine CINTESIS@RISE Faculty of Medicine of the University of Porto Porto Portugal; ^3^ Department of Gastroenterology São João Hospital Center Porto Portugal; ^4^ Clinical Pharmacology Unit São João Hospital University Center Porto Portugal; ^5^ Department of Gastroenterology Vila Nova de Gaia/Espinho Hospital Center Vila Nova de Gaia Portugal

**Keywords:** clinical practice, endoscopy, histology, inflammatory bowel disease, ulcerative colitis

This editorial is in response to “Real‐world uptake of standard indices in the reporting of endoscopy and histology of ulcerative colitis (UC): results of a global survey.”

Endoscopy plays a vital role in the diagnosis, monitoring, and surveillance of UC, with the ultimate goal of achieving endoscopic remission as a treatment target. The significance of histological features in UC was first recognized in 2016 by the Food and Drug Administration through the inclusion of a histological instrument in the definition of mucosal healing.^1^ Over the years, histological activity has become a common tool to predict the prognosis of UC patients, including clinical relapse, need for corticosteroids, colectomy, or hospitalization.^2^, ^3^

Even though the histologic remission (HR) concept varies among reports, two recent independent international panels^4^ defined it as the absence of neutrophil infiltration. While HR is not formally recognized as a treatment target for UC,^5^ the Selecting Therapeutic Targets in Inflammatory Bowel Disease II^6^ guidelines suggest that histological assessment should be performed to evaluate the extent of remission.

In addition, the European Crohn's and Colitis Organisation (ECCO) has recently recommended the use of the Robarts Histopathology Index and Nancy Index for clinical trials; the Nancy Index is recommended for routine clinical practice due to its simplicity.^4^

To assess the implementation rate of current recommendations and gauge the utilization of endoscopic and histologic reporting, Nardone and colleagues conducted a survey involving more than 350 clinicians from 60 countries.^7^ This study provided valuable insights. First, over 90% of the participants reported applying validated endoscopic scores, with the Mayo subscore identified as the preferred index, and most participants (more than 70% for quiescent UC and over 90% for active UC) reported taking biopsies to assess microscopic activity. Still, the reported number of biopsies varied significantly, ranging from 0 to 36. Considering the lack of analysis regarding the timing of endoscopy and specific biopsy locations, this finding emphasizes the need for standardized biopsy protocols (timing, number, and location). Also, the obtained dysplasia surveillance pattern was quite diverse: ∼40% reported using mainly virtual chromoendoscopy, ∼20% dye‐spray chromoendoscopy, and ∼20% preferred white‐light imaging. This practical insight diverges from the ECCO guidelines in which dye spray is still the recommended procedure. However, more recent advice has highlighted virtual chromoendoscopy as the preferred technique since it allows (at least) similar dysplasia detection rates while reducing procedure time.^8^, ^9^, ^10^ In this context, revised European guidelines are awaited. Still in the scope of the survey results, around 60% of the participants recognized the role of histological analysis in assessing disease extension and excluding microscopic activity before treatment de‐escalation; 40% declared to use it for treatment optimization. However, only 22% of the respondents applied histological scores, mostly the Nancy Index (used by two‐thirds of the participants) or the Geboes score. In addition, only 10% were familiar with the cut‐offs used to define remission in the referred indexes. These results suggest that physicians are aware of the prognostic relevance of persistent inflammation but are struggling to objectively monitor it in everyday practice.

Notwithstanding, the external validity of the findings of this survey may be reduced by a few aspects: (i) all respondents were physicians interested in inflammatory bowel disease (IBD); (ii) the proportion of answers from American physicians was low; (iii) around 70% of the physicians were from academic and/or tertiary centers, and one third was from sites with high‐volume of IBD patients; and (iv) half of the centers had a pathologist dedicated to IBD. These features seem to indicate that the unbiased real‐world application of the scores, particularly histological ones, is lower. Indeed, another recent survey from Australia^11^ reached slightly different conclusions when compared with the survey discussed here. Indeed, in this cohort of 89 gastroenterologists (from which, 30% self‐reported as subspecialists in IBD), <8% reported the use of a systematic histologic scoring system and <12% were certain of the proposed remission cut‐offs.

Further efforts are needed to achieve a multidisciplinary consensus on endoscopic and histologic reporting for UC monitoring. This consensus is essential to ensure consistency across hospitals while enhancing communication and improving patient care. In addition, it is crucial to promote formal training in scoring, especially for histology, as the disparity between routine practice and academic expertise may hamper the incorporation of histology as a therapeutic target.^12^ To finalize, the application of a dichotomous ‘neutrophil‐only' index, such as the PICaSSO Histologic Remission Index,^13^ which avoids subjective grading of activity, can enhance the practicality of histological assessment, whether aided by artificial intelligence tools or not.

## AUTHOR CONTRIBUTIONS

Maria Manuela Estevinho was involved in manuscript drafting and editing. Fernando Magro coordinated manuscript drafting and performed a critical revision.

## CONFLICT OF INTEREST STATEMENT

F. Magro served as a speaker and received honoraria from Merck Sharp & Dohme, Abbvie, Vifor, Falk, Laboratórios Vitória, Ferring, Hospira, and Biogen. M.M. Estevinho has no conflicts of interest to disclose.

## Data Availability

Data sharing is not applicable to this article as no new data were created or analyzed in this study.
